# Significant High Lipid Profile in a Woman With Obstructive Jaundice

**DOI:** 10.1210/jcemcr/luad080

**Published:** 2023-07-12

**Authors:** Mashael Albargawi, Ibtihal Abdulaal

**Affiliations:** Adult Endocrinology Department, King Saud Medical City, Riyadh 12746, Kingdom of Saudi Arabia; Adult Endocrinology Department, King Saud Medical City, Riyadh 12746, Kingdom of Saudi Arabia

**Keywords:** obstructive jaundice, lipid, cholesterol, triglyceride

## Abstract

Gallbladder disease is one of the most common gastrointestinal tract diseases. In obstructive jaundice, there is a reduction in bile flow out of the liver secondary to the blocked bile or pancreatic duct, which leads to excess bile and its products accumulating in the blood. One of these products is lipoprotein X (*LpX*); its presence is associated with a lipoprotein pattern characterized by an increased concentration of low-density lipoprotein (LDL) cholesterol. Few published articles have reported the association between obstructive jaundice and hyperlipidemia. This report describes a unique case of a Saudi female patient diagnosed with obstructive jaundice, presenting with extreme hypercholesterolemia, which was reduced significantly 1 week after endoscopic retrograde cholangiopancreatography (ERCP). Correct recognition and investigation of the lipid profile are important for differentiating *LpX*-mediated hypercholesterolemia caused by obstructive jaundice from other causes of elevated LDL concentrations. Differentiation may affect the patient's therapeutic management.

## Introduction

Gallbladder disease is one of the most common diseases of the gastrointestinal tract. Multiple studies have shown an association between gallstone formation and serum lipid level abnormalities [1]. The overall prevalence of gallstones is 6.83% and is more common in females than males. The risk factors for developing gallstones are age 40 years or older, a history of hypertension and familial gallstones, a high waist-to-height ratio of 0.5 or greater, diabetes, thyroid disease, and high C4 levels [2].

Many factors control stone formation, such as cholesterol supersaturation of the secreted bile, bile concentration inside the gall bladder, crystal triglyceride nucleation, and abnormal gall bladder emptying [3]. Cholesterol is insoluble in water, and its solubility in the bile needs adequate bile salts and phospholipids. Elevated cholesterol or reduced phospholipids and bile acid cause multilamellar vesicles to form, causing nucleation of the cholesterol crystals and leading to stone formation [4].

Hypercholesterolemia is common in the clinical setting, but severe elevations of lipid profiles more than 3- or 4-fold are rare. Some causes of these substantial elevations are apolipoprotein E deficiency; homozygous low-density lipoprotein (LDL) receptor deficiency, which is known as familial hypercholesterolemia; hepatic lipase deficiency; and obstetric biliary cholestasis [5].

In obstructive jaundice, there is a reduction in bile flow out of the liver secondary to the blocked bile or pancreatic duct. This blockage inhibits bile drainage from the bloodstream into the intestines, which leads to excess bile and its products accumulating in the blood [6]. Lipoprotein X (*LpX*) is an abnormal unesterified cholesterol and phospholipid-rich lipoprotein particle regurgitating from bile into the bloodstream. Its presence is associated with a lipoprotein pattern characterized by increased LDL cholesterol [7].

Few published articles have reported the association between obstructive jaundice and hyperlipidemia; one was conducted in 1970 for 665 patients with obstructive jaundice, and researchers found that hypercholesterolemia in 435 of the patients was explained by the presence of *LpX* [8].

In 2008, researchers reported a high LDL level of 27.67 mmol/L (1070 mg/dL) in a 45-year-old woman after obstructive jaundice [9]. In 2017, researchers reported another case of *LpX* in a 44-year-old man with a significantly increased LDL level of 58.11 mmol/L (2247 mg/dL) with obstructive biliary cholestasis [5].

Here we describe a unique case of a Saudi female patient diagnosed with obstructive jaundice, presenting with extreme hypercholesterolemia, which was reduced significantly after endoscopic retrograde cholangiopancreatography (ERCP).

## Case Presentation

An 84-year-old Saudi married woman living with her family in ALMozahmyah City was diagnosed with type 2 diabetes 20 years ago. She also had hypertension, dyslipidemia, and hypothyroidism. She was admitted through the emergency room at King Saud Medical City in Riyadh in August 2022 with a history of 1 month of progressively increasing, not-radiating abdominal pain in the right upper quadrant, associated with nausea, general weakness, decreased appetite, and undocumented weight loss. She also noted a history of skin discoloration with itching, pale stool, and dark urine in the previous 2 weeks. The patient was admitted as a having a case of obstructive jaundice for percutaneous transhepatic cholangiography and magnetic resonance cholangiopancreatography (MRCP).

At admission, her medications consisted of insulin glargine 10 units once daily, sitagliptin 100 mg, metformin 1500 mg, amlodipine 5 mg, atorvastatin 20 mg, levothyroxine 75 mcg, and paracetamol 500 mg for abdominal pain. After admission, all oral antihyperglycemic medications stopped. The patient was kept on 18 units of subcutaneous insulin glargine at bedtime with premeal insulin aspart of 8 units; the doses were modified according to the sliding scale.

The patient had no history of smoking, alcohol consumption, new medication, or herbal use. In addition, there was a positive family history of controlled type 2 diabetes and dyslipidemia in her 2 sons, diagnosed after age 45, with no family history of cardiac disease.

## Diagnostic Assessment

On examination, she was afebrile and hemodynamically stable. She is an average-weight person with a body mass index of 18.75. She was jaundiced, and her abdomen was tender, particularly in the upper right quadrant, where the gallbladder was palpable, with a positive Murphy sign. There were no signs of tendon xanthomata, xanthelasma, or premature corneal arcus.

As the patient was referred from another city for the first time, her profile had no previous baseline laboratory results. Therefore, at admission, new blood investigations were ordered, as shown in Table 1; her fasting lipid profile showed a marked elevation of total cholesterol level at 18.76 mmol/L (725.4 mg/dL) and an LDL level at 16.98 mmol/L (656.6 mg/dL). We repeated the lipid profile with some investigations 2 days later to confirm the result and avoid laboratory errors, showing a similar elevation result (see Table 1).

**Table 1.** Blood investigations at admission, 2 days after admission, and 1 week after endoscopic retrograde cholangiopancreatography

Blood test	Normal level/unit	At admission	2 d after admission	1 wk after ERCP
Total cholesterol	<5.17 mmol/L	18.76	15.68	7.31
	<200 mg/dL	725.4	606.3	282.7
LDL	<2.58 mmol/L	16.98	13.57	5.35
	<100 mg/dL	656.6	524.7	206.9
HDL	>1.55 mmol/L	0.72	0.69	1.28
	>60 mg/dL	27.84	26.68	49.5
TGs	<1.7 mmol/L	2.23	3.11	1.48
	<150 mg/dL	197.52	275.47	131.1
Amylase	28-100 IU/L	31	42	30
AST	0-32 IU/L	143	170	51.7
ALT	0-33 IU/L	178	175	36
ALP	35-129 IU/L	682	690	326
GGT	0-39 IU/L	884	1027	728
Total bilirubin	5-20 µmol/L	195	181	36.9
	0.3-1.9 mg/dL	11.40	10.58	2.15
Direct bilirubin	1.7-5.1 µmol/L	175	164	33
	0-0.3 mg/dL	10.23	9.59	1.9
HbA_1c_, %	<5.6% (DCCT)	9.64	—	—
	<114 mg/dL	230	—	—
	<6.3 mmol/L	12.8	—	—

**Abbreviations:** ALP, alkaline phosphatase; ALT, alanine transaminase; AST, aspartate transaminase; DCCT, Diabetes Control and Complications Trial; ERCP, endoscopic retrograde cholangiopancreatography; GGT, γ-glutamyltransferase; HbA_1c_, glycated hemoglobin A_1c_; HDL, high-density lipoprotein; LDL, low-density lipoprotein; TGs, triglycerides.

Abdominal ultrasound demonstrated gallbladder stones with common bile duct and intrahepatic biliary duct dilatation of 1.45 cm and no pericholecystic fluid.

## Treatment

We performed the MRCP, showing 2 gallstones with a mass replacing the gallbladder fossa. The next day the patient underwent ERCP with biliary stenting. The patient tolerated the procedure well and was discharged in good condition with the same insulin doses and oral antihyperglycemic, thyroxin, and oral antihypertensive agents with the replacement of atorvastatin 20 mg by rosuvastatin 20 mg.

## Outcome and Follow-up

One week after discharge, we repeated her fasting lipid profile, showing significant reduction and improvement; her total cholesterol level dropped to 7.31 mmol/L (282.7 mg/dL) and her LDL level to 5.35 mmol/L (206.9 mg/dL), while her triglyceride level was 1.48 mmol/L (131.1 mg/dL) and high-density lipoprotein level was 1.28 mmol/L (49.5 mg/dL). Liver enzymes tests were also repeated and showed improvement, as shown in Table 1. However, a liver core biopsy showed a metastatic adenocarcinoma, suggesting the primary origin of the pancreaticobiliary or upper gastrointestinal tract. The patient was referred to palliative care for follow-up.

## Discussion

We report this case with markedly elevated total cholesterol and LDL levels in a patient with obstructive jaundice because researchers have reported few cases with similar lipid levels in the English-language medical literature.

The liver is a crucial organ in lipid and lipoprotein metabolism; therefore, hepatic diseases usually manifest as disturbances in lipid metabolism [10]. Cholestatic liver diseases are often associated with hypercholesterolemia, explained by an abnormal lipoprotein called LpX, which was isolated and described in the 1960s and 1970s in patients with obstructive jaundice and increasing LDL levels [8, 10].

*LpX*-mediated hypercholesterolemia has been reported to be an early marker of any reduction in intrahepatic or extrahepatic bile flow, with a high concordance of around 95% of cases between its presence and cholestasis. It can also present in the pediatric age group, as reported in the case of an 18-month-old patient diagnosed with abrupt onset of obstructive cholestatic jaundice due to biliary stenosis resembling too high levels of *LpX*-mediated hypercholesterolemia [7].

In all reported cases from the literature, the lipid elevations started after the development of obstructive jaundice. They resolved dramatically in the follow-up laboratory results after surgical intervention [5, 8, 9], indicating that the elevated lipid profile—mainly in LDL—is temporary and secondary to obstructive jaundice. Once this obstruction is managed, the lipid profile drops within weeks of intervention.

We can apply the previous explanation from reported cases to our case; even without a baseline lipid profile before obstructive jaundice presentation, the fast drop in the lipid profile only 1 week after ERCP indicates that the hyperlipidemia improved once the cause was managed.

## Learning Points

- Obstructive jaundice can cause a significant elevation in total cholesterol and LDL levels, which will drop rapidly after obstruction release.
- Screening for lipid profiles in patients with obstructive jaundice is reasonable for detecting abnormally high levels and improving patient outcomes.
- It is important to differentiate *LpX*-mediated hypercholesterolemia caused by obstructive jaundice from other causes of elevated LDL concentrations because this may affect the patient's therapeutic management.

## Contributors

All authors made individual contributions to authorship: I.A. was involved in diagnosing and managing this patient and reviewing this manuscript; M.A. was responsible for reviewing the patient file, writing the case report, and manuscript submission; finally, all authors reviewed and approved the final draft.

## Data Availability

Original data generated and analyzed during this study are included in this published article.

## Abbreviations

ERCP: endoscopic retrograde cholangiopancreatography
LDL: low-density lipoprotein
*LpX*: lipoprotein X
MRCP: magnetic resonance cholangiopancreatography
